# Diabetic cardiomyopathy from a gut microbiota perspective: research progress and prospects

**DOI:** 10.3389/fmed.2025.1737326

**Published:** 2026-01-12

**Authors:** Yuying Liang, Yan Gao, Wei Wang, Zhuo Zhao

**Affiliations:** 1 School of Clinical Medicine, Shandong Second Medical University, Weifang, Shandong, China; 2 School of Qingdao University, Qingdao, Shandong, China; 3 Department of Cardiovascular Surgery, Shandong Public Health Clinical Center, Jinan, Shandong, China; 4 Department of Cardiovascular Medicine, Jinan Central Hospital, Jinan, Shandong, China

**Keywords:** diabetes, diabetic cardiomyopathy, gut microbiota, inflammatory, mitochondria dysfunction

## Abstract

Recent evidence indicates that gut microbiota dysbiosis impairs intestinal barrier function, induces endotoxemia, and consequently triggers systemic inflammatory cascades. Advances in gut microbiota research have further elucidated its critical role in the pathogenesis of diabetes and its complications. This review examines the relationship between diabetic cardiomyopathy and gut microbiota, delineating microbial metabolite-mediated and immunomodulatory influences on myocardial function, while proposing novel therapeutic strategies.

## Pathological mechanism of diabetes cardiomyopathy

In 2017, approximately 451 million adults worldwide had diabetes, a figure projected to rise to 693 million by 2045 (1). Notably, half of all individuals with hyperglycemia are estimated to remain undiagnosed. DCM typically remains asymptomatic during its initial phase. The earliest detectable manifestations include left ventricular hypertrophy and diminished left ventricular compliance, with impaired early diastolic function constituting a core pathophysiological feature (4).

Diabetic cardiomyopathy (DCM)—a prevalent cardiac complication in diabetic patients—is characterized by structural and functional myocardial abnormalities that ultimately progress to heart failure. The molecular pathogenesis of DCM involves interconnected mechanisms including inflammation, hyperglycemia, insulin resistance, fatty acid dysregulation, oxidative stress, mitochondrial dysfunction, and endothelial dysfunction (2). Hyperglycemia directly promotes the accumulation of advanced glycation end products (AGEs) and chronically stimulates the mitochondrial electron transport chain, leading to overproduction of reactive oxygen species (ROS) and consequent oxidative stress. Concurrently, insulin resistance not only compromises glucose uptake and utilization in cardiomyocytes but also disrupts lipid homeostasis, resulting in cardiac ectopic lipid deposition and lipotoxicity. Oxidative stress and lipotoxicity further impair mitochondrial structure and function, inducing mitochondrial dysfunction, which exacerbates ROS generation and perpetuates a self-reinforcing “metabolic–oxidative” vicious cycle.

Of particular significance, inflammation emerges during early-stage diabetes and acts as a key driver of DCM progression (3). These metabolic and oxidative insults at the cellular level activate several pathological signaling cascades. On one hand, ROS and AGEs trigger inflammatory pathways such as nuclear factor kappa B (NF-κB), initiating a low-grade inflammatory response. On the other hand, hyperglycemia and oxidative stress activate the transforming growth factor-β1 (TGF-β1)/SMAD pathway and the renin–angiotensin–aldosterone system (RAAS), promoting cardiac fibroblast activation and extracellular matrix deposition, thereby driving myocardial fibrosis. Furthermore, persistent metabolic stress also induces cardiomyocyte apoptosis and disturbs autophagic balance.

These core mechanisms act synergistically, inducing severe metabolic dysregulation characterized by lipid accumulation (lipotoxicity), progressive mitochondrial impairment, sustained oxidative stress, inflammatory activation, myocardial fibrosis, and enhanced apoptosis. Collectively, these pathological alterations drive progressive deterioration of myocardial contractility, culminating in heart failure. In summary, the cardinal pathological hallmarks of DCM include hyperglycemia, lipotoxicity, oxidative stress, inflammatory cascades, and fibrotic remodeling.

## Relationship between gut microbiota and diabetes cardiomyopathy

As a prevalent complication of diabetes, DCM compromises patient quality of life and increases mortality, while elevating heart failure risk in individuals with pre-existing cardiovascular disease. However, effective strategies for preventing and treating DCM remain limited due to its complex pathogenesis. Extensive studies reveal significant gut microbiota alterations in diabetic patients, characterized by substantial reductions in butyrate-producing bacteria and bile acid-metabolizing species. Concurrently, potentially pathogenic bacteria (e.g., *Salmonella* and *Shigella* spp.) proliferate, establishing a state of gut dysbiosis. Gut dysbiosis promotes metabolic dysregulation in cardiomyocytes, exacerbates pathological processes such as heightened inflammatory responses and mitochondrial dysfunction, ultimately triggering cardiomyocyte hypertrophy and accelerating the progression of DCM. The primary mechanisms involved include:

### Short-chain fatty acids

Short-chain fatty acids (SCFAs), primarily acetate, propionate, and butyrate, are microbial metabolites derived from colonic fermentation of dietary fiber (5). These compounds critically regulate immune function and maintain intestinal barrier integrity (6), with emerging roles in metabolic and cardiovascular homeostasis (7). Mechanistically, elevated plasma propionate levels reduce postprandial insulin secretion (8) and enhance glucose regulation (9). Notably, butyrate attenuates cardiomyocyte apoptosis, suppresses reactive oxygen species (ROS) production, and stimulates angiogenesis (10), while concurrently improving glucose homeostasis and mitigating inflammation—thereby retarding DCM progression.

Butyrate exemplifies these cardioprotective effects. It enhances myocardial function by modulating cardiomyocyte energy metabolism and inflammatory responses via activation of G protein-coupled receptors (GPCRs; e.g., GPR41/GPR43) (11). Butyrate binding to cardiomyocyte GPR43 initiates PI3K/Akt signaling (12), promoting phosphorylation-dependent stabilization of hypoxia-inducible factor 3α (HIF-3α) (13). As a key HIF family member, HIF-3α serves as an endogenous inhibitor that effectively attenuates cardiac hypertrophy and fibrosis by antagonizing hypoxia-inducible factor 1α (HIF-1α) signaling—which otherwise drives pathological cardiomyocyte growth during hypoxia. Under chronic hyperglycemia, HIF-3α suppression upregulates pro-apoptotic proteins (e.g., Caspase-3) while downregulating anti-apoptotic counterparts (e.g., Bcl-2), accelerating cardiomyocyte apoptosis. Critically, reduced butyrate levels correlate with HIF-3α downregulation and hypermethylation at specific CpG sites, implicating this axis in DCM pathogenesis (14). Evidence suggests that reduced HIF3A expression increases the risk of gestational diabetes mellitus (15).

In murine models, butyrate additionally modulates Nucleotide-binding Oligomerization Domain (NOD)-Like Receptors (NLRs)—key regulators of inflammation that maintain tight junction integrity via free fatty acid receptor 2-dependent mechanisms. Butyrate potently suppresses NLRP3 inflammasome activation and signaling. Hyperglycemia potently activates the NLRP3 inflammasome by elevating reactive oxygen species (ROS) production, which upregulates thioredoxin-interacting protein (TXNIP). TXNIP binding to NLRP3 initiates inflammasome assembly (16), leading to caspase-1 activation that cleaves pro-IL-1β and pro-IL-18 into mature cytokines (IL-1β/IL-18). Subsequent release of these pro-inflammatory mediators exacerbates cardiac inflammation (17). Crucially, NLRP3 gene silencing significantly attenuates cardiac inflammation and fibrosis while improving cardiac function in diabetic rats (16). These findings demonstrate butyrate's capacity to mitigate inflammatory responses and cell death in DCM through NLRP3 inflammasome inhibition. Furthermore, the anti-aging protein Klotho and statins (e.g., rosuvastatin) ameliorate DCM-associated cardiac fibrosis, apoptosis, and dysfunction via NLRP3 inflammasome suppression (18).

Butyrate demonstrates additional cardioprotective effects, including blood pressure reduction and attenuation of ischemia-reperfusion injury (19). From a metabolic perspective, fumarate serves as an essential electron acceptor in the biosynthesis of butyrate. Reduced fumarate availability not only impairs butyrate synthesis but also leads to the accumulation of succinate. Functioning as a critical mediator of inflammation and hypoxia signaling, succinate potentiates reactive oxygen species (ROS) generation across tissues during ischemia-reperfusion and exacerbates insulin resistance via mitochondrial ROS overproduction (20). Succinate accumulation further activates three convergent pathways: nuclear factor-kappa B (NF-κB) signaling, hypoxia-inducible factor 1α (HIF-1α) signaling, and the renin-angiotensin-aldosterone system (RAAS). This tripartite activation promotes vascular pathology through smooth muscle cell differentiation, proliferation, migration, and fibrotic remodeling. Therefore, therapeutic strategies aimed at elevating butyrate levels inherently possess the potential to correct succinate accumulation and intervene in the pathological processes of DCM at its source.

Butyrate is an effective inhibitor of histone deacetylases (HDAC). In type 2 diabetic murine models, butyrate—a potent HDAC inhibitor—activates the MKK3/p38/PRAK axis, reversing high-fat diet-induced hypercholesterolemia, glucose intolerance, and metabolic disorders. Consequently, it suppresses cardiomyocyte apoptosis, reduces ROS generation, and stimulates angiogenesis, thereby preventing DCM development (10). Complementary studies in type 1 diabetic mice confirm butyrate-mediated HDAC inhibition attenuates apoptosis, improves ventricular function, mitigates remodeling, suppresses hypertrophy, and ultimately impedes DCM progression. Other SCFA members, such as acetate, also enhance histone acetylation levels and remodel chromatin structure through a similar HDAC inhibition mechanism. This, in turn, upregulates the transcription of insulin signaling-related genes (e.g., GLUT4), thereby improving insulin sensitivity in both cardiac and skeletal muscle (21). Collectively, the SCFA family ameliorates cardiometabolic disturbances in the diabetic context via epigenetic regulation.

### Bile acid metabolism

The gut microbiota critically regulates host metabolic homeostasis through bile acid metabolism modulation. Synthesized from hepatic cholesterol and stored in the gallbladder, bile acids undergo postprandial release to facilitate dietary lipid digestion via emulsification and micelle formation (22). Crucially, gut microbiota-derived secondary bile acids reciprocally regulate microbial composition while modulating host metabolic processes. Bacterial deconjugation of bile acids impairs enterohepatic recirculation, reducing reabsorption efficiency and consequently disrupting lipid digestion. Alternatively, specific secondary bile acids function as signaling molecules that directly influence insulin sensitivity, thereby altering fatty acid utilization and storage dynamics. Excess fatty acids promote ectopic lipid deposition. During accelerated β-oxidation of these lipid reservoirs, substantial reactive oxygen species (ROS) generation induces mitochondrial dysfunction—a key driver in diabetic cardiomyopathy pathogenesis.

TGR5 (G protein-coupled bile acid receptor 1, GPBAR1) is a G protein-coupled receptor primarily activated by bile acids. Ligand binding triggers adenylyl cyclase activation, elevating intracellular cyclic adenosine monophosphate (cAMP) levels. This initiates downstream signaling cascades involving protein kinase A (PKA) and AMP-activated protein kinase (AMPK), which cooperatively suppress pro-inflammatory pathways—notably nuclear factor kappa B (NF-κB) and signal transducer and activator of transcription 3 (STAT3)—thereby attenuating systemic inflammation. Mechanistically, TGR5 regulates cardiomyocyte fatty acid uptake by modulating CD36 activity (23). Specifically, TGR5 inhibits DHHC4-mediated palmitoylation of CD36, reducing its plasma membrane translocation. This suppression of membrane trafficking decreases fatty acid uptake, ameliorating myocardial lipid accumulation and pathological remodeling—identifying a novel therapeutic axis for diabetic cardiomyopathy. CD36, a transmembrane fatty acid transporter (24), exhibits gut microbiota-dependent regulation. Intriguingly, cardiac sarcolemmal CD36 overexpression occurs in obese rats without pathological stimuli (25), confirming its role in metabolic dysregulation. CD36 deficiency substantially increases cecal microbial alpha-diversity with enriched *Bacteroides, Rikenella*, and *Prevotella* genera (26). These butyrate-producing taxa enhance microbiota stability and ecological resilience while maintaining glucose homeostasis. This finding reveals a direct mechanistic link between bile acid/TGR5 signaling and butyrate production. The TGR5-CD36 axis not only directly regulates myocardial lipid metabolism but may also modulate the host's metabolic state, thereby shaping a gut microbiota enriched with butyrate-producing bacteria. The resulting butyrate subsequently delivers systemic cardioprotective benefits, as described earlier. Consequently, the TGR5-CD36 axis emerges as a pivotal hub that integrates bile acid signaling with the salutary effects of short-chain fatty acids, underscoring the “gut-heart axis” as a functionally interconnected network rather than a set of discrete pathways.

CD36 overactivation disrupts cardiac lipid homeostasis by uncoupling fatty acid uptake from oxidation, resulting in lipid accumulation, lipotoxicity, and consequent cellular dysfunction. Experimental evidence confirms that the synthetic TGR5 agonist INT-777 confers cardioprotection and enhances myocardial function through targeted receptor activation (23). By engaging TGR5 signaling, INT-777 reduces cardiac lipid deposition, attenuates cardiomyocyte lipotoxicity, and impedes DCM progression—demonstrating significant therapeutic potential. Notably, bile acids deoxycholic acid (DCA) and taurocholic acid (TCA) similarly improve cardiac function via TGR5 pathway activation, effectively reducing lipid accumulation. Mechanistic studies reveal that DCA, TCA, INT-777, and related agonists share a conserved action: TGR5 activation inhibits CD36 palmitoylation and membrane translocation, thereby normalizing lipid metabolism and cardiac performance. Consequently, pharmacological targeting of the TGR5-CD36 axis represents a novel therapeutic paradigm for modulating aberrant lipid transport in DCM.

Beyond facilitating lipid digestion and absorption, bile acids activate the Farnesoid X Receptor (FXR)—a ligand-dependent nuclear receptor transcription factor that orchestrates bile acid, lipid, and glucose homeostasis (27). The semi-synthetic chenodeoxycholic acid derivative obeticholic acid (OCA) functions as a potent FXR agonist. Through synergistic crosstalk with the Nrf2 pathway, FXR activation mediates: Metabolic dysregulation amelioration, Oxidative stress and inflammation attenuation, Myocardial fibrosis and cardiac dysfunction suppression (28). However, it is important to note that the clinical application of OCA is constrained by side effects such as pruritus and elevated LDL-cholesterol, which may limit its use in certain patient populations. Therefore, while OCA represents a promising therapeutic candidate, its translational potential warrants careful evaluation in the context of safety and tolerability.

### TMAO

Trimethylamine N-oxide (TMAO) biosynthesis originates from gut microbial metabolism of dietary precursors including choline and L-carnitine. Specific enzymatic complexes (e.g., choline TMA-lyase CutC/D) convert these substrates into trimethylamine (TMA), which enters systemic circulation. Hepatic flavin-containing monooxygenase 3 subsequently oxidizes TMA to TMAO. Elevated TMAO accumulation correlates significantly with diabetic cardiomyopathy pathogenesis. Mechanistically, TMAO impairs cardiomyocyte contractile function and disrupts intracellular calcium handling—primarily through induction of mitochondrial dysfunction that reduces cellular energy production (28).

Gut microbiota dysbiosis and increased intestinal permeability drive chronic inflammation, which directly activates the Nuclear Factor Kappa-B (NF-κB) signaling pathway (29, 30). Trimethylamine N-oxide (TMAO) potentiates this pathway by stimulating NADPH oxidase-mediated hydrogen peroxide (H_2_O_2_) overproduction. NF-κB upregulation in endothelial cells alters key enzymes regulating vasoactive mediators—notably reducing endothelial nitric oxide synthase (eNOS) expression and activity, thereby decreasing nitric oxide (NO) bioavailability (31). This NO deficiency heightens cardiomyocyte sensitivity to intracellular Ca^2^^+^, increasing myocardial stiffness and impairing diastolic function. Furthermore, NF-κB induces vasoactive factors including interleukin-6 (IL-6) and intercellular adhesion molecule 1 (32), which promote endothelial dysfunction and accelerate diabetic cardiomyopathy progression.

Thioredoxin-interacting protein (TXNIP) mediates crosstalk between oxidative stress and inflammation by activating the NLRP3 inflammasome under trimethylamine N-oxide (TMAO) stimulation (33). Mechanistically, TMAO suppresses sirtuin 3 expression and superoxide dismutase 2 activity, augmenting mitochondrial reactive oxygen species (mtROS) accumulation. Elevated mtROS promotes TXNIP dissociation from thioredoxin (TRX), ablating its antioxidant function (34). The liberated TXNIP translocates to mitochondria or endoplasmic reticulum, directly binding NLRP3 to trigger inflammasome assembly and caspase-1 activation. Caspase-1—a cysteinyl aspartate-specific protease—forms active dimers that catalyze pro-inflammatory cytokine maturation and induce pyroptosis (programmed inflammatory cell death), thereby accelerating diabetic cardiomyopathy progression.

Both *in vivo* and *in vitro* studies have revealed that TMAO promotes cardiac hypertrophy and fibrosis via the Smad3 signaling pathway. The TMA synthesis inhibitor 3,3-dimethyl-1-butanol (DMB) has been found to prevent cardiac hypertrophy and fibrosis by modulating the transforming growth factor-β1 (TGF-β1)/Smad3 and p65 nuclear factor-κB (NF-κB) signaling pathways (35). This finding thereby confirms the role of TMAO in ventricular remodeling. While extensive observational studies support a strong association between TMAO and DCM, and interventional studies using inhibitors such as DMB suggest a causal role, further research is still required to definitively establish this causality in humans.

**Figure 1:**
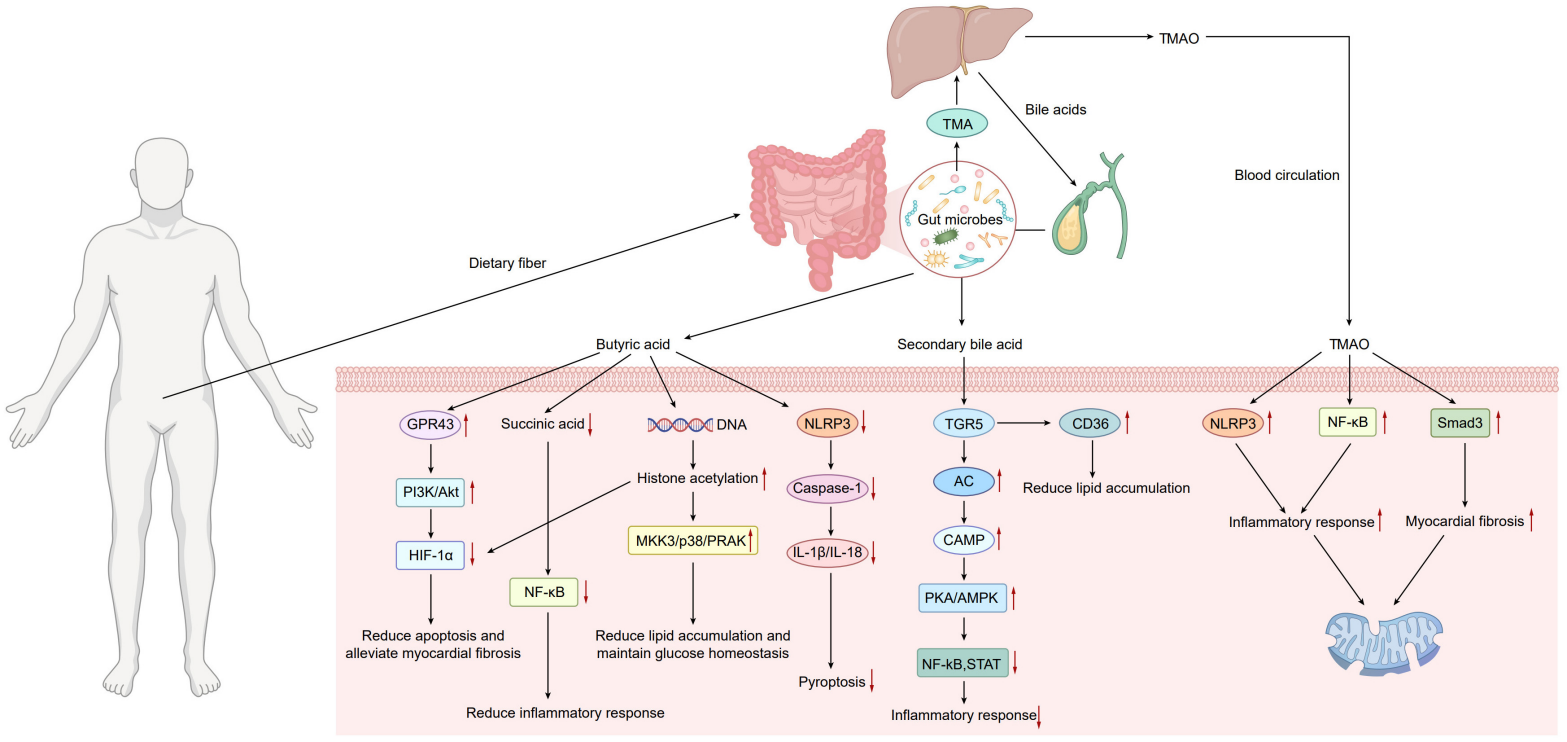
Dietary fiber is metabolized by gut microbiota to produce substances such as butyrate, bile acids, and TMA. TMA is subsequently oxidized to TMAO in the liver, which then enters the systemic circulation. Butyrate binds to GPR43, activating the PI3K/Akt pathway and inhibiting the HIF-1α signaling pathway, thereby reducing apoptosis and attenuating cardiac fibrosis. Butyrate reduces succinate accumulation, suppressing inflammatory signaling pathways such as NF-κB and mitigating inflammatory responses. As an HDAC inhibitor, butyrate reduces lipid accumulation through activation of the MKK3/p38/PRAK pathway. Bile acids can bind to TGR5, leading to increased intracellular cyclic AMP (cAMP) levels. This activates downstream signaling pathways, including protein kinase A (PKA) and AMPK. Consequently, this inhibits pro-inflammatory pathways such as NF-κB and STAT3, alleviating inflammation. Additionally, TGR5 modulates CD36 activity, thereby regulating lipid metabolism. TMAO activates the NLRP3 inflammasome and induces inflammatory responses via NF-κB inflammatory signaling. TMAO also activates the Smad3 pathway, exacerbating cardiac fibrosis, and causes mitochondrial damage.

In summary, elevated TMAO levels are positively associated with the development of DCM. TMAO contributes to the progression of DCM through four major pathways: impairing mitochondrial function/energy metabolism, activating systemic inflammation (NF-κB, NLRP3), promoting fibrosis (Smad3), and inducing endothelial dysfunction. Furthermore, increased TMAO levels have been identified as an independent predictor of poor prognosis in patients with diabetic cardiomyopathy (Figure 1).

## Therapeutic perspectives

Given the role of the gut microbiota in diabetic cardiomyopathy, modulating the gut microbiota may emerge as a novel therapeutic strategy for future management of this condition. Below are several potential therapeutic directions:

### Synergistic management of probiotics, prebiotics, and drug safety

Probiotic supplementation has been demonstrated to reduce markers of inflammation and oxidative stress. Modulating the gut microbiota composition through the administration of probiotics (e.g., *Akkermansia muciniphila*) or prebiotics (e.g., dietary fiber) increases the abundance of beneficial bacteria and suppresses the proliferation of harmful bacteria. This leads to improvements in glucose and insulin metabolism (36, 37), thereby alleviating the inflammatory state. However, when considering incorporating the regulation of gut microbiota into the comprehensive management of DCM, attention must also be paid to the safety of conventional clinical medications. In clinical practice, many patients with diabetic cardiomyopathy (DCM) require myocardial perfusion imaging or similar assessments due to coexisting coronary artery disease. These procedures often involve the administration of vasodilators such as Regadenoson to evaluate myocardial blood flow. It is noteworthy that a recent large-scale real-world study based on the FDA Adverse Event Reporting System revealed that Regadenoson may induce neurological and urinary adverse effects not listed in its prescribing information, in addition to known reactions such as chest pain and dyspnea (38). This finding suggests that when using such agents in diabetic patients, the potential for additional risks should be fully considered and integrated with gut microbiota modulation strategies to enable safer and more personalized treatment.

Inulin—a naturally occurring water-soluble dietary fiber and prebiotic formally recognized as dietary fiber by the U.S. FDA (21 CFR 101.9)—is widely incorporated into functional foods due to its demonstrated health benefits. Experimental studies establish that inulin modulates gut microbiota composition in high-fat diet-induced diabetic models and reduces hyperglycemia. Human trials further confirm its efficacy in lowering postprandial glucose and ameliorating insulin resistance. Mechanistically, inulin suppresses pro-inflammatory cytokines (e.g., IL-6, TNF-α), thereby attenuating systemic inflammation, preserving mitochondrial function, and inhibiting diabetic cardiomyopathy progression (39). A recent meta-analysis demonstrates that inulin-type fructans improve lipid-glucose metabolism through multi-faceted mechanisms: (i) favorable gut microbiota remodeling; (ii) enhanced gut hormone secretion (iii) reduced systemic inflammation; and (iv) improved intestinal barrier integrity (40).

*Lactobacillus plantarum* confers cardioprotection by enhancing myocardial antioxidant capacity through upregulation of cardiac antioxidant enzymes [e.g., superoxide dismutase, glutathione peroxidase (GPx)]. It significantly downregulates key apoptosis markers—including tumor necrosis factor-α (TNF-α), Fas ligand (FasL), and cleaved caspases-3/8/9—thereby attenuating cardiomyocyte apoptosis. Furthermore, this probiotic ameliorates diabetic cardiomyopathy progression by improving systemic glucose-lipid homeostasis (41).

Lactic acid bacteria (LAB) maintain intestinal homeostasis by enriching beneficial microbiota (e.g., *Lactobacillus, Bifidobacterium*) while suppressing pathogenic colonization. Specific probiotic strains—including *Lactobacillus reuteri* and *L. curvatus*—modulate cytokine networks by downregulating pro-inflammatory mediators (TNF-α, IL-6) and upregulating anti-inflammatory factors (IL-10) (42). Concurrently, LAB enhance cardiac antioxidant defenses through superoxide dismutase (SOD) and glutathione peroxidase (GPx) activation, mitigating oxidative cardiomyocyte damage. Experimental evidence reveals synergistic anti-inflammatory and cardioprotective effects when LAB combine with dapagliflozin and crocin, mediated through PPARγ pathway activation (43). This underscores the critical importance of preserving host-microbiota mutualism. Targeted modulation of probiotics and prebiotics thus represents a viable strategy to ameliorate diabetic cardiomyopathy progression.

### Fecal microbiota transplantation

Fecal microbiota transplantation (FMT)—a therapeutic intervention transferring healthy donor microbiota to restore gut ecological balance—enhances insulin sensitivity and modulates metabolism in diabetic patients. Its application for diabetic cardiomyopathy warrants further investigation. Mechanistically, FMT preserves cardiac structure and function by: (i) downregulating cardiac L-type amino acid transporter 1 expression; (ii) reducing branched-chain amino acid accumulation; and (iii) suppressing mTOR/Bax/Bcl-2/caspase-3 signaling activation. Crucially, FGF21 knockdown abolishes these cardioprotective effects, confirming FGF21 as the essential mediator of FMT-induced cardiac improvement (44).

FMT offers a novel therapeutic approach for cardiomyopathy by remodeling the “gut-heart axis” through metabolic modulation (suppressing TMAO, and elevating SCFAs) and immunoregulation. However, its clinical translation requires overcoming three major bottlenecks: ecological niche mismatch, individual heterogeneity, and long-term safety concerns. Future development necessitates integrating omics-microbiome-transcriptomics strategies (OMT), engineered bacterial technologies, and continuous monitoring systems to advance the precision implementation of personalized microbiota-based therapies in cardiovascular medicine.

### Metabolic product intervention

Pharmacological or dietary modulation of gut microbiota-derived metabolites—particularly suppressing trimethylamine N-oxide (TMAO) production while enhancing short-chain fatty acid (SCFA) generation—represents a viable strategy for improving myocardial function in diabetic cardiomyopathy. Experimental evidence demonstrates that myricetin ameliorates cardiac dysfunction and myocardial fibrosis by repairing intestinal barrier integrity through enhanced microbial diversity and reduced mucosal permeability (45). Dietary interventions require restriction of red meat consumption, especially processed meats, as primary sources of L-carnitine and choline. In omnivorous individuals, gut microbiota metabolizes L-carnitine into pro-atherogenic TMAO at significantly higher rates than in vegetarians (46). Conversely, dietary fiber—notably soluble fiber (e.g., β-glucans) and resistant starch—serves as essential microbial substrates for SCFA production. We propose regular consumption of prebiotic-rich foods combined with physical activity to maintain gut microbial homeostasis and mitigate DCM progression.

### Metabolomics and causal inference propose precise interventions for the treatment of DCM

Elucidating the causal relationship between gut microbiota and their metabolites and diabetic cardiomyopathy (DCM) is pivotal for translating microbiome research into clinical applications. In recent years, genetic causal inference methods, particularly Mendelian randomization (MR), have emerged as powerful tools to address this challenge. For instance, a large-scale MR study analyzing 1,400 blood metabolites systematically identified causal associations between various lipid and bile acid metabolites and the risk of abdominal aortic aneurysm, providing an important paradigm for mechanistic investigation and target discovery in metabolic cardiovascular diseases (47). Future research should leverage this methodology, utilizing large-scale genomic and metabolomic databases to rigorously examine the genetic causality between key gut-derived metabolites—such as short-chain fatty acids (SCFAs), bile acids, and trimethylamine N-oxide (TMAO)—and the development of DCM. This approach will help distinguish genuine pathogenic drivers from mere epiphenomena, thereby identifying the most promising precision therapeutic targets and generating high-level evidence to support individualized treatment strategies based on the gut–heart axis.

## Conclusion

The gut microbiota plays a pivotal regulatory role in the onset and progression of diabetic cardiomyopathy (DCM). This influence is mediated primarily through its metabolites—such as short-chain fatty acids (SCFAs), bile acids, and trimethylamine N-oxide (TMAO)—and its immunomodulatory network, which collectively affect myocardial metabolism, inflammation, and fibrosis, thereby forming a complex pathophysiological link known as the gut-heart axis. Although current research has begun to reveal the close association between gut dysbiosis and DCM—along with some underlying molecular mechanisms—and has pointed to potential therapeutic strategies such as probiotics, fecal microbiota transplantation, and metabolite-targeted interventions, translating these findings into clinically effective treatments remains challenging.

Most existing evidence comes from animal models and observational studies, which, while strongly suggestive of correlation, still require validation through prospective cohort studies and more refined interventional trials to establish direct causality between specific gut microbiota changes and the pathogenesis of DCM in humans. The composition of the gut microbiota varies greatly among individuals due to genetic, dietary, environmental, and medication-related factors. This heterogeneity may lead to instability in microbiota-based diagnostic biomarkers and inconsistent efficacy of generalized therapeutic strategies, highlighting the need for future research to move toward personalized, precision-focused investigations of host–microbiome interactions.

In summary, research into the gut microbiota has opened a revolutionary new perspective on the prevention and management of DCM. Its ultimate clinical value will be realized through the precise dissection, intervention, and management of the gut–heart axis. Only by deeply integrating macroscale microbial ecology with microscale molecular mechanisms, alongside individual-specific characteristics, can we truly usher in an era of microbiota-based precision medicine for diabetic cardiomyopathy and deliver meaningful benefits to patients.
